# Long-term clinical efficacy of topical treatment with recombinant human nerve growth factor in neurotrophic keratopathy: a novel cure for a rare degenerative corneal disease?

**DOI:** 10.1186/s13023-022-02236-6

**Published:** 2022-02-16

**Authors:** Alice Bruscolini, Marco Marenco, Giuseppe Maria Albanese, Alessandro Lambiase, Marta Sacchetti

**Affiliations:** Department of Sense Organs, Sapienza University of Rome, Viale del Policlinico 155, 00169 Rome, Italy

**Keywords:** Neurotrophic keratopathy, Cenegermin, Recombinant human nerve growth factor eye drop solution, Corneal sensitivity, Visual acuity, Tear function

## Abstract

**Background:**

Neurotrophic keratopathy (NK) is a rare, degenerative ocular disease characterized by reduction or loss of corneal sensitivity and development of non-healing corneal epithelial defects and ulcers. Cenegermin, a recombinant human nerve growth factor (rhNGF) eye drop solution, is the first drug approved for the treatment of NK. The aim of our study is to evaluate the long-term efficacy of this innovative topical treatment in patients with NK.

**Methods:**

Retrospective, consecutive, observational case series study from a single-center setting (Department of Sense Organs, University Sapienza of Rome, Rome, Italy). 18 patients with diagnosis of stage 2 or 3 NK, treated with Cenegermin 20 mcg/ml eye drops were followed for up to 48 months. Recurrence of lesion during follow-up was evaluated at 12, 24, 36, and 48 months. In addition, corneal sensitivity, Schirmer tear test, and visual acuity (VA) were recorded at baseline, end of treatment, and at 12, 24, 36, and 48 months.

**Results:**

Three patients experienced recurrence of persistent epithelial defects (PEDs) within 12 months and one patient experienced recurrence of a corneal ulcer within 36 months. Corneal sensitivity was significantly improved at all timepoints (*P* < 0.05). Significant improvements in visual acuity and tear production were seen at the completion of treatment as well as at 12, 24, and 36 months (*P* < 0.05) when compared to baseline.

**Conclusions:**

A single 8-week treatment regimen of Cenegermin eye drops has clinical efficacy that can persist for up to 48 months. The long-term clinical utility of treatment with Cenegermin for NK was demonstrated through the low rate of lesion recurrence along with improvements in corneal sensitivity and tear production.

## Background

Damage to the ophthalmic branch of the trigeminal nerve that innervates the cornea can lead to neurotrophic keratopathy (NK), a rare, progressive disease characterized by loss of sensation, decreased tear production, epithelial breakdown, and impaired corneal healing [1, 2]. NK can be classified into 3 stages based on the severity of the corneal damage: mild stage 1—epithelial alterations, moderate stage 2—non-healing persistent epithelial defect (PED) and severe stage 3—corneal ulcer [3]. If left untreated, NK can progress to corneal perforation, scarring, and profound vision loss. Even with conventional treatment, NK can recur after a period of healing, which makes early and adequate treatment important to preventing both progression and recurrence [1].

Conventional management of NK has previously been limited to use of artificial tears at earlier disease stages, while more severe, refractory cases were typically addressed by surgical approaches. However, these conventional treatments often leave patients at risk of recurrence as they do not address the underlying nerve damage that is the basis of NK [4]. Thus, novel promising treatments for NK able to restore long-term corneal innervation and sensitivity are being developed.

Nerve growth factor (NGF) is known to be involved in the regulation of growth, maintenance, proliferation, and survival of certain sympathetic and sensory neurons [5]. In the healthy cornea a two-way interaction occurs between epithelial cells and the corneal nerves. In NK patients, reduced corneal sensation and decreased tear production make the corneal surface vulnerable to injuries and impair its ability to heal [2]. Disturbing these mutual nerve-epithelium interactions can lead to development of persistent epithelial defects. Increasing experimental and clinical evidence have shown that nerve growth factor (NGF) plays an important role in corneal Pathophysiology supporting its use in NK, although its exact role is not entirely clear [5–8]. These studies led to the development of cenegermin, a recombinant human nerve growth factor (rhNGF) [Dompé Farmaceutici SpA, Milan, Italy], that has been approved for topical ophthalmic use by both the EMA and FDA [9–11]. Cenegermin was studied in two, randomized, double-masked, vehicle-controlled pivotal phase 2 studies (NGF0212/REPARO and NGF0214) that evaluated its efficacy and safety in patients with moderate-to-severe NK [11, 12].

The REPARO trial showed that a statistically significant proportion of patients achieved complete corneal healing after one 8 week course of cenegermin treatment (72.0% vs 33.3% of vehicle treated patients) [11]. Similar results were seen in NGF0214 which found that 65.2% of cenegermin-treated patients achieved complete healing compared to 16.7% in the vehicle group [12]. The REPARO trial followed patients for 1 year and found that more than 96% of patients treated with cenegermin who achieved complete healing did not experience a recurrence [11]. In the NGF0214 trial, 12.5% of patients who achieved complete healing after masked cenegermin treatment experienced recurrence and underwent a second course of cenegermin treatment. All of these patients healed after retreatment and remained healed through the end of the 24-week follow-up period [12].

In addition to evaluating corneal healing, the clinical trials for cenegermin also assessed corneal sensitivity, reflex tearing, and visual acuity as a secondary efficacy endpoints. Although not statistically significant, in both studies patients treated with rhNGF exhibited greater improvement in corneal sensitivity (measured directly by Cochet-Bonnet aesthesiometer) from baseline to weeks 4 and 8 than the vehicle-treated control patients. Additionally, there was no statistically significant change in visual acuity between the cenegermin- and vehicle-treated groups in these trials, but there was a trend toward improvement favoring cenegermin. In both studies, corneal sensitivity, tear production, and visual acuity were not measured beyond 8 weeks [11, 12].

Other studies conducted with cenegermin have shown similar corneal healing trends to those shown in the clinical trials. A prospective, observational study by Mastropasqua et al. included 18 cenegermin-treated patients with NK and compared them to 20 healthy age- and sex-matched control subjects [13]. The response to cenegermin was evaluated using corneal fluorescein staining, Schirmer tear test, and corneal sensitivity testing with nerve morphology analyzed by in vivo confocal microscopy. This study showed complete corneal healing after 8 weeks of treatment in all patients and a significant improvement in tear production, but also a significant increase in mean nerve density, nerve branch number, and nerve fibre diameter compared to baseline [13]. This study showed that cenegermin helps improve the structure and function of corneal nerves, which indicates that cenegermin may help to resolve the underlying etiology of NK thereby healing the cornea and preventing recurrence. However, this study did not include a follow-up period to evaluate efficacy beyond the 8-week treatment period.

While the pivotal clinical trials and real world case reports have demonstrated the clinical utility of cenegermin for the treatment of NK, these studies offered limited long term efficacy data beyond 1 year [11, 12]. Further long term evaluations for clinically relevant efficacy measurements such as corneal sensitivity, tear production, or visual acuity would also be helpful in assessing ocular surface health [14]. Therefore, this retrospective, consecutive, observational case series was designed to evaluate the clinically significant efficacy endpoints for up to 48 months after a single 8-week treatment regimen with cenegermin in patients with moderate-to-severe NK.

## Methods

This long-term follow-up report was a retrospective, consecutive, observational single center (Department of Sense Organs, University Sapienza of Rome, Rome, Italy), case series of patients treated with cenegermin ophthalmic solution (Oxervate®) 6 times daily for 8 weeks and then followed for up to 48 months. The medical records of patients diagnosed with stage 2 or 3 NK and treated with cenegermin between dates January 15, 2015 and July 18, 2018 were reviewed for data on disease recurrence, visual acuity, corneal sensitivity, and tear production. This study was approved by the Investigational Review Board (IRB) of University Sapienza of Rome, Italy (IRB number 5338) with informed consent obtained at follow-up visits.

Adult patients (≥ 18 years of age) with NK were diagnosed with stage 2 (persistent epithelial defect (PED)) or stage 3 (corneal ulcer) disease based on Mackie classification. The main inclusion criteria were: i). diagnosis of stage 2 or 3 NK, ii). topical treatment with cenegermin eye drops for 8 weeks and iii). at least 24 months of follow-up available. Patients were not allowed concomitant topical ophthalmic medications or other previously applied treatments during the duration of cenegermin treatment. During the entire follow-up period, use of artificial tears were permitted as needed. In case of recurrence, retreatment with cenegermin was not permitted and patients were treated with conventional management based on clinical findings including therapeutic contact lens application as first choice, followed by amniotic membrane transplantation and/or tarsorrhaphy in refractory cases. Patients with recurrence did not exit the study at the time of recurrence.

Efficacy assessments were evaluated at baseline, end of treatment (8 weeks), 12 months, 24 months, 36 months, and 48 months. Corneal healing, defined as less than 0.5-mm fluorescein staining (the lower limit of reliable slit-lamp assessment) in the lesion area, was assessed by the investigator at week 8 and during follow-up period. Recurrence was evaluated over the previous year at each follow-up time point. Recurrence was defined as return of PED (i.e. stage 2) or corneal ulcer (i.e. stage 3) following initial corneal epithelial healing. Additional evaluations of corneal sensitivity, tear production, and BCDVA were performed. BCDVA was measured by Snellen chart and expressed as a decimal unit. Corneal sensitivity was measured using a Cochet-Bonnet aesthesiometer (CBA) (Luneau Ophthalmologie, Chartres, France) directly within the corneal lesion and in each quadrant outside of the lesion. Tear production was assessed by the Schirmer test I without topical anesthesia.Schirmer strip (NewTech SPA, Milan, Italy) were placed in the lower tear lake and the length of the moistened portion of the strip, after 5 min, was measured. No safety data were collected.

Statistical analysis was performed with IBM SPSS Statistics V22.0 software using paired t-test to compare observations at each follow-up to those at baseline.

## Results

Eighteen patients were included in this analysis, all of which had data for up to 24 months of follow-up. Additionally, 10 patients had data available for up to 36 months of follow-up and 9 patients had data available for up to 48 months of follow-up. Of the 18 patients, 11 patients with stage 2 NK and 7 patients with stage 3 NK were included in the study. Seven male and 11 female patients ranged in age from 46 to 83 years old with a mean age of 65.3 ± 12.6 years. The most common underlying etiologies included herpes simplex virus (HSV) keratopathy (n = 7, 38.8%) and Sjogren’s Syndrome (n = 6, 33.3%) (Table 1).

**Table 1:** Demographic and clinical features at baseline

Eye treated	N
Right eye	7
Left eye	11
**NK stage**	
Stage 2	11
Stage 3	7
**Age (years)**	
Range	46–83
Mean ± SD	65.3 ± 12.6
**Gender (n)**	
Male	7
Female	11
**NK etiology (n)**	
HSV keratitis	7
Sjogren Syndrome	6
Multiple ocular surgeries	2
Diabetes mellitus	1
Ocular cicatricial pemphigoid	1
Ocular chemical burn	1
**Other associated diseases**	
Cataract	
Lymphoma	1
Calcific band keratopathy	1
	1

HSV = herpes simplex virus; NK = neurotrophic keratitis; SD = standard deviation

**Fig. 1:**
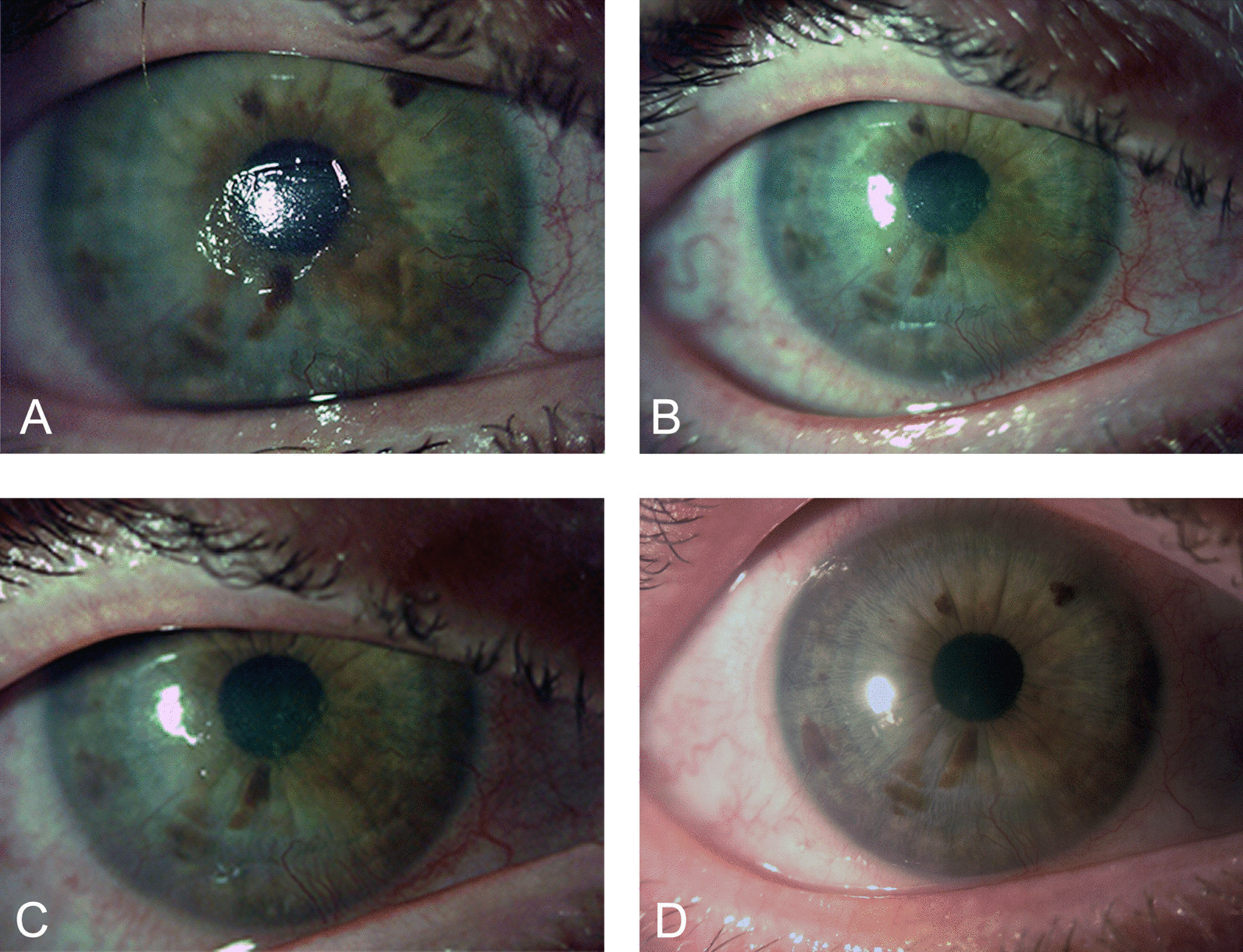
Representative slit -lamp pictures showing the clinical progression of neurotrophic keratopathy after cenegermin treatment. **A** central, neurotrophic corneal lesion at baseline, **B** corneal lesion completely healed after 8 weeks of treatment, **C** corneal healing persisted at 12 months of follow up, and **D** after 24 months of follow up

All patients presented with corneal healing at the end of cenegermin treatment (week 8). At each follow-up visit, the patients were assessed for recurrence of NK, as classified by stage 2 or 3 based on the reports of neurotrophic PED or ulcers in the patients’ charts over the year of the follow-up period (Fig. 1). As seen in Table 2, out of 18 patients, only three had recurrence of PEDs (stage 2) during the first year of follow-up and one patient had recurrence of a neurotrophic corneal ulcer (stage 3) during the third year of follow-up. All patients with recurrence of NK were treated with therapeutic contact lens application and topical lubricants and all showed complete corneal healing.

**Table 2:** Recurrence at each follow-up visit

	Stage 2 recurrence	Stage 3 recurrence
End of treatment (n = 18)	0	0
12 months (n = 18)	3	0
24 months (n = 18)	0	0
36 months (n = 10)	0	1
48 months (n = 9)	0	0

**Fig. 2:**
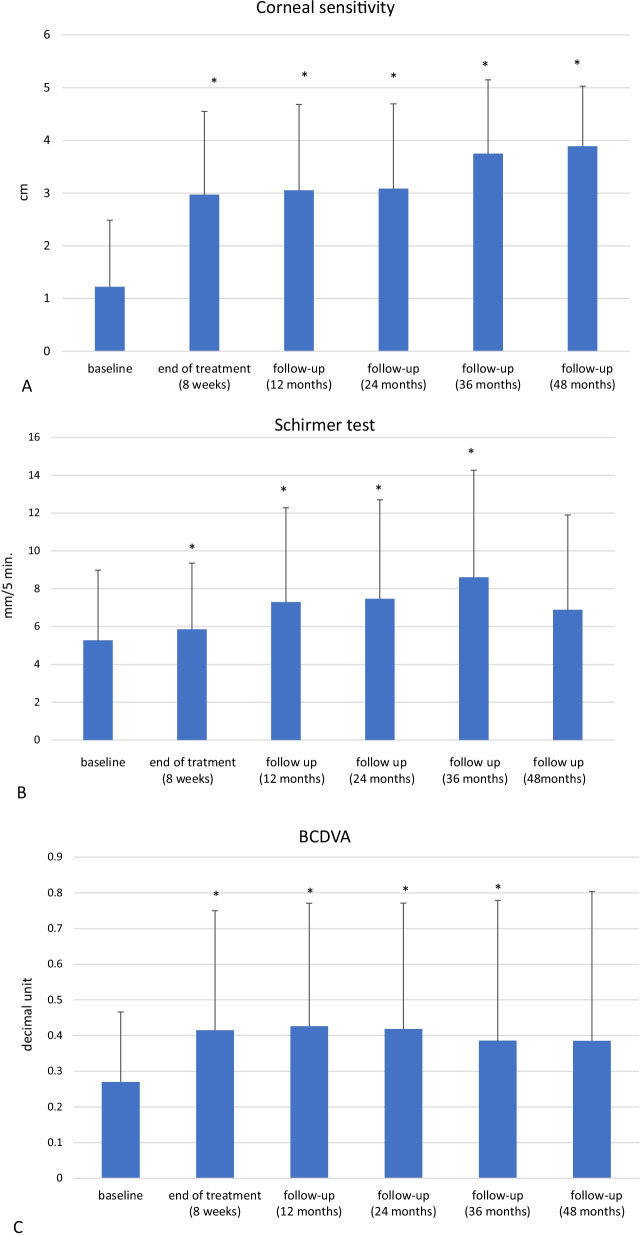
Bar graph showing the long-term improvement in clinical outcome of neurotrophic keratopathy after cenegermin treatment. **A** There was a significant improvement in corneal sensitivity from baseline to end of treatment and at follow-up (at 12, 24, 36, and 48 months). **B** There was a significant improvement in tear production (Schirmer test) from baseline to end of treatment and at follow-up (at 12, 24, and 36 months). **C** There was a significant improvement in best corrected distance visual acuity (BCDVA) from baseline to end of treatment and at follow-up (at 12, 24, and 36 months). *P < 0.05

Corneal nerve damage, which causes NK, results in partial or total loss of corneal sensation, therefore assessing changes in corneal sensation could be a reflection of changes in corneal nerves. We wanted to determine if these patients had improved corneal sensitivity after cenegermin treatment, and if this continued overtime. Patients had a mean corneal sensation of 1.2 ± 1.3 cm (measured with a Cochet-Bonnet Aesthesiometer [CBA]) at baseline. As shown in Fig. 2A we found that cenegermin treatment considerably enhanced corneal sensitivity as early as by end of the 8 week treatment. Interestingly, these improvements persisted throughout 48 months of follow-up. The mean change from baseline to end of treatment was 1.75 ± 0.81 cm (*P* < 0.05; 95% CI, 1.35 cm to 2.15 cm) with continuous improvement during the course of the follow-up period over time, with 1.83 ± 0.84 cm (*P* < 0.05; 95% CI, 1.42 cm to 2.25 cm) at 12 months, 1.86 ± 0.76 cm (*P* < 0.05; 95% CI, 1.48 cm to 2.24 cm) at 24 months, 1.95 ± 0.83 cm (*P* < 0.05; 95% CI, 1.36 cm to 2.54 cm) at 36 months, and finally 2.11 ± 1.08 cm (*P* < 0.05; 95% CI, 1.28 cm to 2.94 cm) at 48 months.

Corneal nerves also elicit protective reflexes that promote tear production [2, 15]. Figure 2B, demonstrates that tear production was significantly improved from a baseline Schirmer’s test of 5.3 ± 3.7 mm after cenegermin treatment and continued to improve during follow-up. The observed mean change from baseline to end of treatment was 0.59 ± 0.94 mm/5 min (*P* = 0.020; 95% CI, 0.11 mm/5 min to 1.07 mm/5 min). During the follow-up period statistically significant improvements persisted at 12 months (2.03 ± 2.02 mm/5 min; *P* = 0.001; 95% CI, 0.99 mm/5 min to 3.07 mm/5 min), 24 months (2.21 ± 2.17 mm/5 min; *P* = 0.001; 95% CI, 1.09 mm/5 min to 3.32 mm/5 min), and 36 months (2.55 ± 2.06 mm/5 min; *P* = 0.001; 95% CI, 1.08 mm/5 min to 4.02 mm/5 min). The mean change at 48 months was not significantly different from baseline (0.61 ± 4.58 mm/5 min; *P* = 0.699; 95% CI, 2.91 mm/5 min to 4.13 mm/5 min).

If left untreated, NK can progress and lead to profound vision loss [1]. This stresses the importance of evaluating visual acuity as a clinically relevant outcome in patient with NK. Mean visual acuity was 0.3 ± 0.2 (measured by the Snellen chart and expressed as decimal unit) at baseline and showed a significant improved after treatment and during follow-up (Fig. 2C). Mean change from baseline to end of treatment was 0.15 ± 0.20 (*P* = 0.008; 95% CI, 0.04 to 0.25). Statistically significant improvements persisted at 12 months (0.16 ± 0.22; *P* = 0.008; 95% CI, 0.05 to 0.27), 24 months (0.15 ± 0.23; *P* = 0.015; 95% CI 0.03 to 0.26), and 36 months (0.23 ± 0.26; *P* = 0.037; 95% CI, 0.02 to 0.44). At 48 months however, the observed improvement was not statistically significant (0.21 ± 0.32; *P* = 0.131, 95% CI, 0.08 to 0.50).

## Discussion

The main purpose of our study is to expand on the previously published data on corneal healing after 8-week treatment of cenegermin and recurrence rates in patients with neurotrophic keratopathy (NK). This study also sought to provide evidence on the long-term effect of cenegermin on additional clinically relevant endpoints including corneal sensitivity, reflex tear production, and visual acuity.

Neurotrophic keratopathy is a progressive disease that leads to recurrent breakdowns of the ocular surface and despite various medical and surgical therapies, NK remains yet challenging to treat.In fact conventional treatments including use of topical lubricants, serum drops, bandage contact lenses, amniotic membranes transplantation and tarsorrhaphy showed variable healing rates in NK and few data are available on their long-term efficacy..Specifically small studies reported a recurrence rate 1- months after AMT treatment ranging from 18 to 46% [16, 17].In addition, in many cases these conventional treatments often do not address the underlying cause of NK but rather only promote corneal healing [1, 4]. In our study, we observed maintenance of corneal healing throughout the 48 months of follow-up in most patients. Furthermore, we also observed continuous and sustained improvement in corneal sensitivity and tear production, which is consistent with the results of another study we have recently published in dry eye patients [18]. The long-lasting effects of cenegermin on corneal sensitivity and tear function in patients with NK suggest it acts by directly targeting the corneal innervation that underlies NK pathogenesis and provide evidence to support the proposed mechanism of action of cenegermin.

While visual acuity does not necessarily reflect the severity of the disease or the healing status, it is a clinically relevant parameter, as NK can potentially lead to impaired visual acuity if not treated properly. In our study, treated patients presented with significantly improved Best corrected distant visual acuity (BCDVA) at 12, 24 and 36 months. At 48 months, BCDVA improvement did not reach statistically significant values, probably due to the lower number of patients.

A limitation to our study was the uncontrolled design and a relatively small number (n = 18) of patients included. However, NK is a rare disease making larger, randomized, placebo-controlled, clinical trials with long follow-up periods difficult to conduct.

## Conclusions

The results of this study demonstrated that long-term one 8 week course of cenegermin was effective in restoring ocular surface homeostasis in terms of corneal epithelial stability, tear film production and corneal sensitivity recovery. This newly found and evidenced continuous efficacy of this treatment opens the door for deeper investigation of both the underlying molecular mechanisms and the potential for larger, long term, prospective, controlled clinical trials.

## Data Availability

The datasets used and/or analysed during the current study are available from the corresponding author on reasonable request.

## Abbreviations

NK: Neurotrophic Keratopathy
NGF: Nerve Growth Factor
rhNGF: Recombinant Human Nerve Growth Factor
PED: Persistent Epithelial Defect
EMA: European Medicines Agency
FDA: Food and Drug Administration
CBA: Cochet-Bonnet Aesthesiometer
BCDVA: Best Corrected Distant Visual Acuity
IRB: Investigational Review Board

## References

[1] Sacchetti M, Lambiase A (2014). Diagnosis and management of neurotrophic keratitis. Clinical ophthalmology (Auckland, NZ).

[2] Muller LJ, Marfurt CF, Kruse F, Tervo TM (2003). Corneal nerves: structure, contents and function. Exp Eye Res.

[3] Dua HS, Said DG, Messmer EM, Rolando M, Benitez-Del-Castillo JM, Hossain PN (2018). Neurotrophic keratopathy. Prog Retin Eye Res.

[4] Mastropasqua L, Massaro-Giordano G, Nubile M, Sacchetti M (2017). Understanding the pathogenesis of neurotrophic keratitis: the role of corneal nerves. J Cell Physiol.

[5] Lambiase A, Sacchetti M, Bonini S (2012). Nerve growth factor therapy for corneal disease. Curr Opin Ophthalmol.

[6] Lambiase A, Rama P, Bonini S, Caprioglio G, Aloe L (1998). Topical treatment with nerve growth factor for corneal neurotrophic ulcers. N Engl J Med.

[7] Bonini S, Lambiase A, Rama P, Caprioglio G, Aloe L. Topical treatment with nerve growth factor for neurotrophic keratitis. Ophthalmology. 2000;107(7):1347–51; discussion 51–2. 10.1016/s0161-6420(00)00163-9 10889110

[8] Lambiase A, Mantelli F, Sacchetti M, Rossi S, Aloe L, Bonini S (2011). Clinical applications of NGF in ocular diseases. Arch Ital Biol.

[9] Ferrari MP, Mantelli F, Sacchetti M, Antonangeli MI, Cattani F, D'Anniballe G (2014). Safety and pharmacokinetics of escalating doses of human recombinant nerve growth factor eye drops in a double-masked, randomized clinical trial. BioDrugs : clinical immunotherapeutics, biopharmaceuticals and gene therapy.

[10] Bonini S, Lambiase A, Rama P, Filatori I, Allegretti M, Chao W (2018). Phase I trial of recombinant human nerve growth factor for neurotrophic keratitis. Ophthalmology.

[11] Bonini S, Lambiase A, Rama P, Sinigaglia F, Allegretti M, Chao W (2018). Phase II randomized, double-masked, vehicle-controlled trial of recombinant human nerve growth factor for neurotrophic keratitis. Ophthalmology.

[12] Pflugfelder SC, Massaro-Giordano M, Perez VL, Hamrah P, Deng SX, Espandar L (2020). Topical recombinant human nerve growth factor (cenegermin) for neurotrophic keratopathy: a multicenter randomized vehicle-controlled pivotal trial. Ophthalmology.

[13] Mastropasqua L, Manuela L, Dua HS, D'Uffizi A, Di Nicola M, Calienno R, et al. In vivo evaluation of corneal nerves and epithelial healing after treatment with recombinant nerve growth factor for neurotrophic keratopathy. American journal of ophthalmology. 2020. 10.1016/j.ajo.2020.04.036 32387431

[14] Di Zazzo A, Varacalli G, Mori T, Coassin M. Long-term restoration of corneal sensitivity in neurotrophic keratopathy after rhNGF treatment. Eur J Ophthalmol. 2020:1120672120953343. 10.1177/1120672120953343 32854535

[15] Mantelli F, Nardella C, Tiberi E, Sacchetti M, Bruscolini A, Lambiase A. Congenital corneal anesthesia and neurotrophic keratitis: diagnosis and management. Biomed Res Int. 2015;2015:805876. 10.1155/2015/805876 PMC4588028 26451380

[16] Kruse FE, Rohrschneider K, Volcker HE (1999). Multilayer amniotic membrane transplantation for reconstruction of deep corneal ulcers. Ophthalmolog.

[17] Sacchetti M, Komaiha C, Bruscolini A, Albanese GM, Marenco M, Colabelli Gisoldi RAM (2021). Long-term clinical outcome and satisfaction survey in patients with neurotrophic keratopathy after treatment with cenegermin eye drops or amniotic membrane transplantation. Graefes Arch Clin Exp Ophthalmol.

[18] Sacchetti M, Lambiase A, Schmidl D, Schmetterer L, Ferrari M, Mantelli F (2020). Effect of recombinant human nerve growth factor eye drops in patients with dry eye: a phase IIa, open label, multiple-dose study. Br J Ophthalmol.

